# Solvates and Polymorphs of Axitinib: Characterization and Phase Transformation

**DOI:** 10.3390/molecules29194696

**Published:** 2024-10-04

**Authors:** Yinhu Pan, Tong Xiao, Yan Wang, Zhiying Pan, Shichao Du, Fumin Xue

**Affiliations:** School of Pharmaceutical Sciences (Shandong Analysis and Test Center), Qilu University of Technology (Shandong Academy of Sciences), Jinan 250014, Chinawangyan57@tju.edu.cn (Y.W.);

**Keywords:** axitinib, crystallization, solvates, phase transition, polymorph

## Abstract

Axitinib (AXTN) is an oral tyrosine kinase inhibitor for the treatment of early to advanced renal cell carcinoma. In this work, solvates of AXTN were prepared in five solvents and subjected to desolvation treatment. The crystal form A of AXTN can form solvates in acetonitrile, DMF, acetic acid, acetic acid + water, and methanol. Different ratios of AXTN and acetic acid will form different products (solvate or directly crystallized into another crystal form (form IV)). The characterization results of thermal analyses confirmed the types of the five solvates. The obtained solvates were desolvated using methods of solid-phase desolvation (heating, exposure to solvent steam, microwave) and solvent-mediated phase transformation (SMPT). The desolvated solids were characterized by PXRD, TGA, DSC, FT-IR, and SEM, and it was ultimately inferred that a new crystal form (form Z) of AXTN could be formed after desolvation. In addition, the solvates obtained in this work experienced mutual transformation via SMPT, which depends on the type of solvents or mixed solvents. The phase transformations of different solid forms were summarized. This study is instructive for exploring solvates and polymorphs of AXTN and understanding phase transitions under different environments.

## 1. Introduction

**Figure 1:**
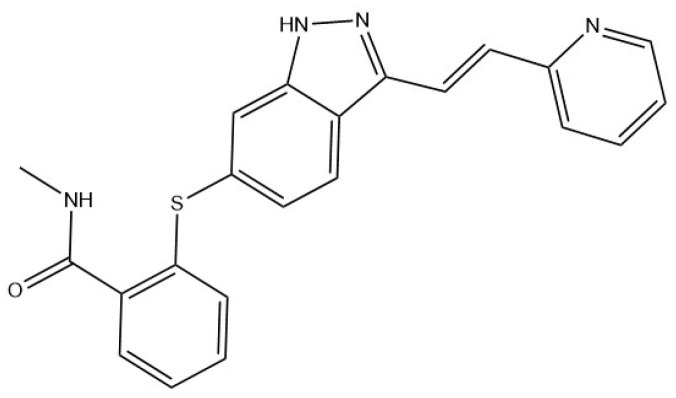
The molecular structure of AXTN.

Axitinib (AXTN, C_22_H_18_N_4_OS, CAS NO.319463-51-9, Figure 1) is an oral tyrosine kinase inhibitor developed by Pfizer in the United States for the treatment of early to advanced renal cell carcinoma [1,2,3,4,5,6,7,8,9]. It can inhibit tyrosine kinase receptors, including vascular endothelial growth factor receptors (VEGFR-1, VEGFR-2, and VEGFR-3), and can be used for renal cell carcinoma (RCC) by inhibiting VEGF-mediated endothelial cell proliferation and survival [10,11,12,13,14,15,16]. In practical applications, some physical and chemical properties of AXTN may affect its efficacy. As an anticancer drug belonging to class II of the Biopharmaceutics Classification System (BCS), AXTN exhibits low solubility (0.2 μg·mL^−1^ at pH = 7.8). This low solubility affects the oral bioavailability of AXTN [17,18,19,20].

Different polymorphs, hydrates, and solvates exhibit different properties, such as density, hygroscopicity, stability, mechanical properties, solubility, and bioavailability [21,22,23,24]. When solvents and drugs are mixed, different interactions occur between their molecules, including surface adsorption, liquid encapsulation, and defect adsorption. If solvent molecules are incorporated into drug molecules during lattice construction, solvates will be formed. Morris KR et al. first classified solvates by structure in 1999 [25], including solvates with independent sites, channel-type solvates, and ion-associated solvates. The difference in crystal structure is actually a reflection of the difference in interaction forces, so the interaction forces between solvent molecules and solute molecules of these kinds of solvates are also different. Generally, for solvates with independent sites, there are interactions between solvent molecules and solute molecules within the crystal lattice, but there are no strong interactions between solvent molecules. As a result, this type of solvate tends to undergo a sharp endotherm when the solvent is removed, and there is a sharp endothermic peak in the DSC curve caused by desolvation. The rate of mass loss is also relatively fast during this period. In the crystal structure of channel-type solvates, solvent molecules are arranged in a chain along a certain direction to form a certain channel structure [21,26]. Some channel solvates can expand their overall unit cell size to absorb non-stoichiometric solvents, termed extended channels [27]. The characteristic of the channel-type solvates in the DSC curve is the wide endothermic peak of the desolvation process. The weight loss process of the desolvation process in the TGA curve is also very slow, and the starting temperature of the desolvation process is generally lower than the boiling point of the solvent. For ion-associated solvates, metal cations are generally present. Metal ions and solvent molecules form complexes through hydrogen bonds. The complexes generally have a higher desolvation temperature.

In terms of AXTN, multiple polymorphic forms (I, IV, VI, XXV, XLI, and crystalline forms of reference code VUSDIX07), two hydrates, over 60 solvates, and multiple salts have been reported [28]. Although some crystal forms of AXTN have been discovered, problems such as the poor solubility of AXTN have not been well solved. Exploring the new solid form of AXTN provides the possibility to solve the problems encountered in its practical application. Ren et al. improved the apparent solubility of AXTN by preparing multi-component crystals of AXTN [29]. They prepared salt-cocrystal of AXTN with fumaric acid and bimolecular cocrystal of AXTN with succinic acid and trans cinnamic acid, improving the apparent solubility and dissolution rate of AXTN without affecting the physical stability of the drug. Qu et al. prepared various solvates of AXTN and theoretically characterized their solvation behavior, analyzing the crystal structure and molecular properties of AXTN solvates [30].

The above studies were conducted on the cocrystal and solvation behavior of AXTN crystal form IV. AXTN can form a variety of solvates, so it can be used as a medium to explore the transformation of AXTN polymorphs. This study investigates the solvation conversion of AXTN crystal form A [31], and new solvates are obtained. A solid-state solvent removal method is provided for preparing a new crystal form of AXTN. By generating some solvates and then desolvating them, the crystal form is transformed, and a new crystal form (form Z) is obtained.

## 2. Results and Discussion

### 2.1. Characterization of Solvates

#### 2.1.1. PXRD Analysis

**Figure 2:**
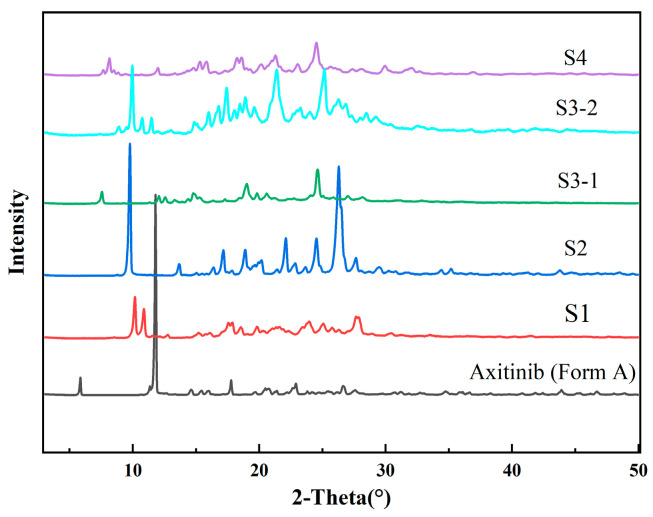
PXRD patterns of AXTN raw materials and solvates.

The PXRD patterns of the solvates of AXTN are shown in Figure 2. Among them, S1–S4 represent solvate of AXTN-acetonitrile, solvate of AXTN-DMF, solvate of AXTN-acetic acid, and solvate of AXTN-methanol, respectively. Figure 2 shows that the PXRD patterns of different solvates are significantly different. For S2 and S3–1, these solvates have been reported in the literature [30] (CSD code: QARVAK and QARVIS), and each has the same PXRD pattern and TGA pattern. The comparison of the PXRD patterns of the solvates obtained in this work and the existing solvates is shown in Figure S1 of the Supplementary Materials.

#### 2.1.2. Thermal Analysis

**Figure 3:**
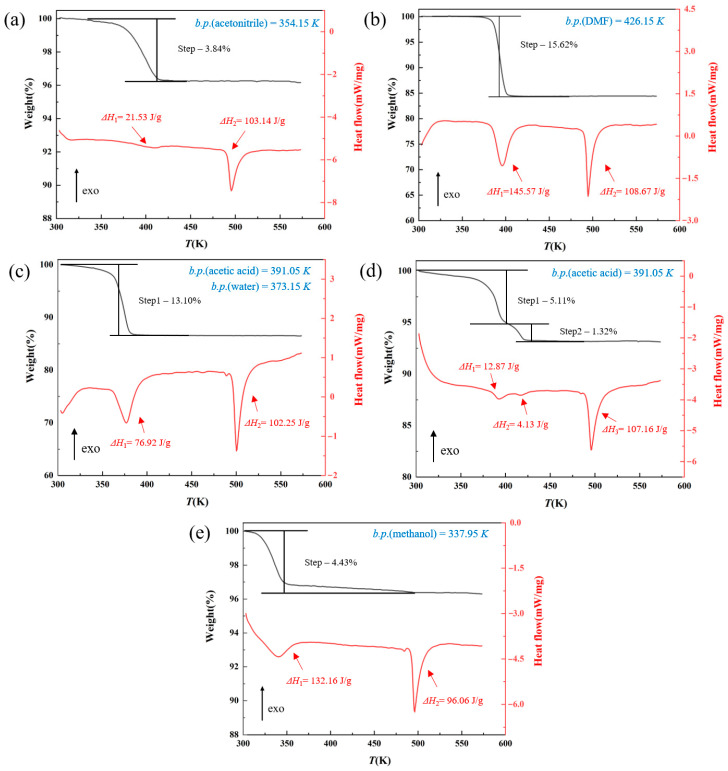
TGA and DSC curves of solvates of AXTN: (**a**) AXTN-acetonitrile solvate (S1); (**b**) AXTN-DMF solvate (S2); (**c**) AXTN-acetic acid-H_2_O solvate (S3–1); (**d**) AXTN-acetic acid solvate (S3–2); (**e**) AXTN-methanol solvate (S4). The heat values represented by the endothermic peak are marked with arrows.

The TGA and DSC curves of the solvates of AXTN are shown in Figure 3. The enthalpy values are marked at the corresponding positions (rough estimate). The ratio of AXTN to solvent in the solvate can be calculated by the weight loss of the sample during heating (b.p. is the expected boiling point of the solvent).

TGA characterization shows that the five solvates exhibit varying degrees of weight loss due to the evaporation of solvent. From the weight loss stages, it can be determined that the ratio of AXTN to solvent for the five solvates is as follows: AXTN/acetonitrile = 3:1; AXTN/DMF = 1:1; AXTN/acetic acid/H_2_O = 2:3:1.5 (reference to CSD code: QARVIS); AXTN/acetic acid = 2:1; AXTN/methanol = 2:1. The two peaks in each DSC curve represent the endothermic processes of solvent removal and melting, respectively. A small endothermic peak appears at about 480 K before the main endothermic peak in the methanol solvate. It indicates that the crystalline form obtained after removing the solvent may undergo a polymorph phase transition (possibly converted to Form A).

The types of the five solvates can be roughly determined from the thermal analysis. The TGA and DSC curves of S2 and S3–1 (Figure 3b,c) show similar phenomena (sharp endothermic peaks and rapid mass loss), corresponding to solvates of independent sites. S3–2 shows two weight loss steps. The first step shows that the weight loss has already occurred at the beginning of heating. The temperature at this time is relatively low (300 K). Combined with the characteristics of different solvate structures, the analysis may show the removal of solvent in the channel-type structure at this time. The second step begins to appear at 400 K. The weight loss occurs at relatively high temperatures, and the weight loss in the second segment is faster, which is consistent with the characteristics of independent-site-type solvates [32,33]. S3–2 may have both channel and independent site structures. The remaining solvates may be channel-type solvates.

In addition, some modern computational methods (such as density functional theory (DFT)) are widely used to evaluate the stability of different forms of drugs, which can be used to verify experimentally obtained crystal structures and predict new crystal structures, understand crystallization and dissolution processes, develop new polymorphic synthesis methods and preparation technologies, etc. [34]. For example, Dubok et al. used DFT to calculate the relative stability of pyrazinamide polymorphs and explored the phase transition mechanism of pyrazinamide through DFT calculations [35]. The DFT calculations can be applied to explain the mechanism of the formation of the reported crystal forms in this work after we solve the single crystal structures in the future.

#### 2.1.3. FT-IR Analysis

The infrared spectra of the solvates of AXTN are shown in Figure S2. Compared with raw materials (AXTN form A), the formation of solvates causes a shift in the position of the characteristic peaks of the corresponding functional groups. The bands at 2960–2970 cm^−1^ of S1, S2, and S3–2 are assigned to the C−H stretching vibration of the solvent molecules (acetonitrile, DMF, and acetic acid). The peak at 1675 cm^−1^ of S2 is assigned to the C=O stretching vibration of the solvent molecules (DMF). The C=O peak observed in acetic acid solvate at 1705 cm^−1^. Compared with the raw material, the peak at 3205 cm^−1^ of S4 is assigned to the O−H stretching vibration of the solvent molecules (methanol).

#### 2.1.4. Crystal Morphology

The surface morphology of the solvates of AXTN was observed using SEM and is shown in Figure S3. The crystals of S1 show flake-like shape (Figure S3a). S2 has a similar flake-like morphology with a shorter aspect ratio (Figure S3c). S3–2 displays agglomerated particles composed of rod-like crystals (Figure S3e). Crystals in the form of S4 grew to be elongated plate-like shape (Figure S3g). SEM images show that although the microstructures of the four solvates are not the same, their surfaces are relatively flat, and there are no inherent pores or cracks.

### 2.2. Desolvation of AXTN Solvates

Table 1 lists the processing methods of different solvates and the crystal forms obtained after this processing.

#### 2.2.1. Solid-Phase Desolvation

**Figure 4:**
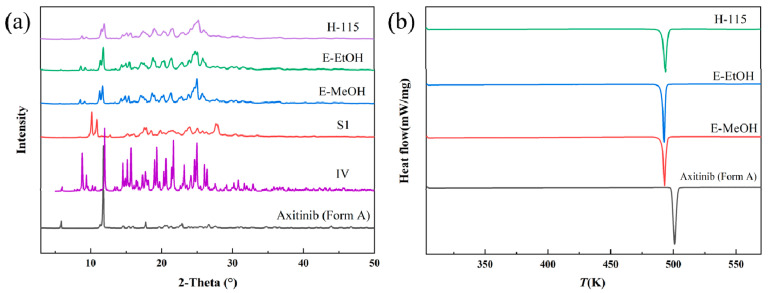
Characterization of S1 and the desolvated products: (**a**) PXRD patterns and (**b**) DSC curves.

Solid-phase desolvation of S1. The PXRD patterns in Figure 4a show that the solvate of AXTN-acetonitrile (S1) transformed to crystal form IV via different solid-phase desolvation treatments (heating at 115 °C, exposure to steam of methanol, exposure to steam of ethanol). It can be confirmed by the DSC curves, which show the endothermic peaks for the melting process of newly formed solids appearing at the same temperature (Figure 4b). As is shown in the SEM image (Figure S3b), the morphology of the product (crystal form IV) changed after methanol-steam-mediated phase transformation. There are pores on its surfaces, which contributed to the evaporation of solvent.

**Figure 5:**
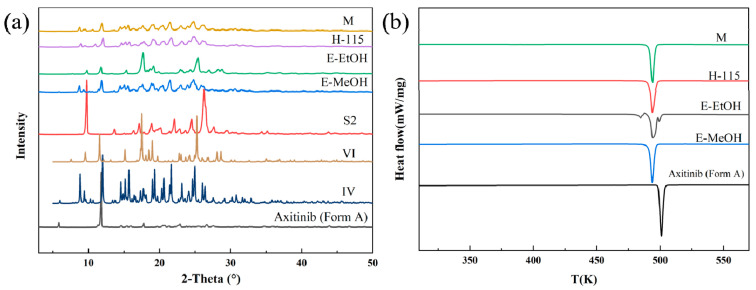
Characterization of S2 and the desolvated products: (**a**) PXRD patterns and (**b**) DSC curves.

Solid-phase desolvation of S2. The PXRD patterns in Figure 5a show that the solvate of AXTN-DMF (S2) transformed into three crystal forms after different solid-phase desolvation treatments. The crystal form IV was obtained after heating at 115 °C, exposure to methanol steam, and microwave treatment. The crystal form VI was obtained after exposure to ethanol steam. This is confirmed by the endothermic peaks in the DSC curves (Figure 5b). The thermal analysis also indicates that the stability of the crystal form VI is not as good as that of the crystal form IV. The crystal form VI may undergo phase transformation at the location where the first endothermic peak appears. The third small endothermic peak roughly coincides with the melting peak of the raw material (form A), suggesting that the crystal form VI may have partially transformed into the crystal form A. After the solid-phase transition in an atmosphere containing methanol vapor, pores also appeared on the crystal surface (form IV) (Figure S3d).

**Figure 6:**
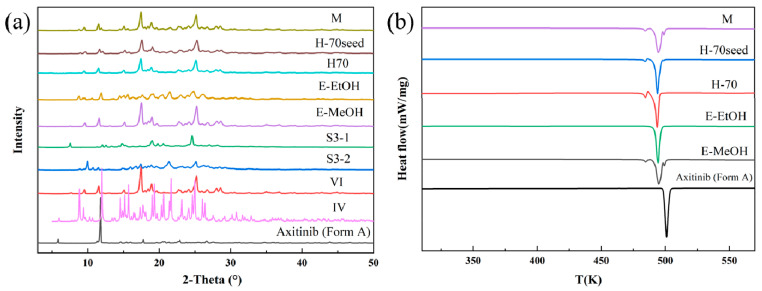
Characterization of S3–2 and the desolvated products: (**a**) PXRD patterns and (**b**) DSC curves.

Solid-phase desolvation of S3–2. The solvate of AXTN-acetic acid (S3–2) transformed into different crystal forms after different solid-phase desolvation treatments (Figure 6a). Specifically, the crystal form VI was created through exposure to methanol steam, heating at 70 °C, heating at 70 °C with seed induction, and microwave treatment. The crystal form IV was obtained through exposure to ethanol steam. From the DSC curves (Figure 6b), the product obtained from S3–2 after microwave treatment and methanol-steam-mediated treatment underwent crystal transformation at around 480 K. This transformation resulted in the formation of crystal form IV and a certain amount of crystal form A. It can be observed that the largest endothermic peak coincides with the melting peak of crystal form IV, and the third peak coincides with the melting peak of raw material. The treatment of heating solvates at 70 °C did not show a third endothermic peak. The samples may have transformed from crystal form VI to crystal form IV. We hope to induce the transformation of solvates into other crystal forms through seeding. However, in the acetic acid system, the addition of seeds (form A) during heating and solvent removal has little effect on the polymorphic transition. The crystal surfaces of the desolvated product also present pores or cracks (Figure S3f).

**Figure 7:**
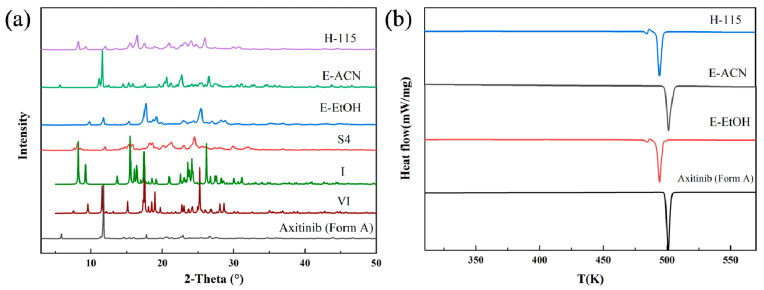
Characterization of S4 and the desolvated products: (**a**) PXRD patterns and (**b**) DSC curves.

Solid-phase desolvation of S4. The PXRD patterns (Figure 7a) show that the AXTN-methanol solvate (S4) converted to three crystal forms after different solid-phase desolvate treatments. They are crystal form A (formed through exposure to acetonitrile steam), crystal form VI (formed through exposure to ethanol steam), and crystal form I (obtained through heating at 115 °C). This is verified by the endothermic peak in the DSC curves (Figure 7b). The thermal analysis also shows that crystal form A is very stable, while both crystal forms I and VI undergo a certain degree of polymorph transformation during the heating process (Figure 7b). It was observed from the SEM image (Figure S3h) that pores also appeared on the surface of the product of AXTN-methanol solvate after acetonitrile-steam-mediated treatment.

#### 2.2.2. Solvent-Mediated Phase Transformation (SMPT)

**Figure 8:**
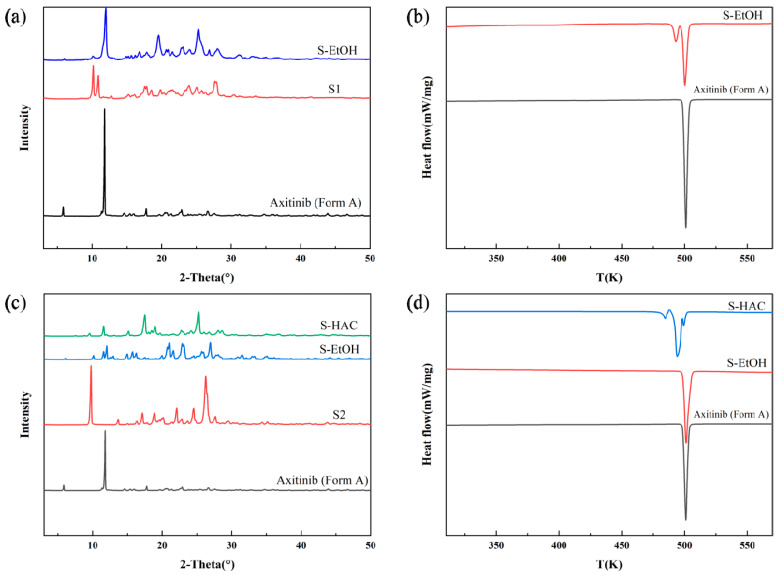
(**a**) PXRD patterns of SMPT of S1; (**b**) DSC curves of SMPT of S1; (**c**) PXRD patterns of SMPT of S2; (**d**) DSC curves of SMPT of S2.

The solvate of AXTN-acetonitrile converted to the new crystal form Z through ethanol-mediated phase transformation. The PXRD pattern of the product showed the appearance of several distinct new peaks (2θ = 19.45°, 23.00°, 25.29°, and so on) (Figure 8a). It can be seen from the DSC curve that the form Z undergoes a polymorph transformation at 490 K (accompanied by the appearance of an endothermic peak), possibly transforming into crystalline form A. In the DSC curve, the second endothermic peak of the product is consistent with that of the raw material (Figure 8b). The TGA curve of form Z is presented in the Supplementary Materials (Figure S6).

After SMPT experiments on AXTN-DMF solvates, two crystal forms can be obtained: crystal form A (formed through ethanol-mediated transformation) and crystal form IV (formed through acetic-acid-mediated transformation). Based on PXRD (Figure 8c) and DSC (Figure 8d) analyses, there also exists some form A in the products.

### 2.3. Phase Transition of AXTN Solvates

#### 2.3.1. Transformation of AXTN Solvates into New Crystal Forms

**Figure 9:**
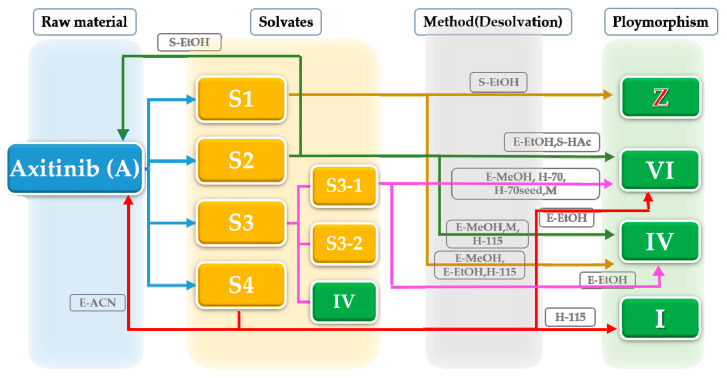
AXTN solvate undergoes phase transition through desolvation.

In summary, the AXTN (form A) can form solvates with acetonitrile, DMF, acetic acid, and methanol. When AXTN is mixed with acetic acid, different ratios of solute and solvent will yield different solid forms. Solid characterization indicates that these solvates can transform into other crystal forms after desolvation. The relationship between the transformation methods and corresponding crystal forms of AXTN is illustrated in Figure 9.

#### 2.3.2. Transformation between Solvates of AXTN

The solvates formed in this work can not only obtain new crystal forms through desolvation, but also transform into another solvate. The PXRD patterns of these solvates are shown in Figure S4. The transformation results between the solvates of AXTN are listed in Table 2 and Figure S5. The conversion between solvates of AXTN is mainly achieved through solvent-mediated transformation. This work also attempted to prepare solvates of AXTN in mixed solvents. If the raw material of AXTN is placed in a mixed solvent with a volume ratio of 1:1, it tends to generate a fixed solvate. For example, placing the raw material of AXTN in a mixed solvent with a volume ratio of acetonitrile to methanol of 1:1 produces the AXTN-methanol solvate. The same applies when DMF/methanol = 1:1 and acetic acid/methanol = 1:1. When other solvates transform into AXTN-acetic acid solvates, the resulting product depends on the ratio of the solvate to the acetic acid solvent. This corresponds to the situation when preparing acetic acid solvate.

## 3. Materials and Methods

### 3.1. Materials

AXTN was supplied by Shandong New Era Pharmaceutical Co., Ltd. (Weihai, China). The organic solvents used in the experiment ware methanol, ethanol, N,N-dimethylformamide (DMF), acetonitrile, and acetic acid. The deionized water was prepared in our laboratory (Arium Advance EDI, Sartorius, Göttingen, Germany). All of the above organic solvents were obtained from Sinopharm Chemical Reagent Co., Ltd. (Shanghai, China), and had a mass fraction purity higher than 0.99. All chemical substances were directly used to prepare the solvates of AXTN without further processing. More detailed information can be found in Table S1 of the Supplementary Materials.

### 3.2. Preparation of Solvates

AXTN-acetonitrile (S1, 3:1) was prepared by slurry method. We added 5 g of AXTN and 60 mL of acetonitrile into the jacketed glass container and used a stirring paddle at a speed of 200 rpm. During the experiment, a thermostatic water bath (CF 41, Julabo, Seelbach, Germany; precision: ±0.05 K) was used to maintain the temperature at 40 °C, which lasted for 6 h before cooling to 20 °C. A solvate of AXTN-acetonitrile (S1) can be collected by filtering the suspension and then drying at room temperature. AXTN-DMF (S2, 1:1, also known as “S6” in the literature [30]) and AXTN-methanol (S4, 2:1) can also be formed in the same way by replacing acetonitrile with DMF and methanol.

AXTN-acetic acid solvate was prepared by slurry method. It should be noted that there are significant differences in the PXRD patterns of the obtained products when the ratio of AXTN to acetic acid is changed. When excess AXTN was mixed with acetic acid and water (1:1, *v*/*v*), the AXTN-acetic acid-H_2_O solvate (S3–1, AXTN: acetic acid: H_2_O = 2:3:1.5, also known as “S2” in the literature [30]) was formed. Suspensions of 2 g of AXTN and 15 mL of acetic acid resulted in the formation of another solvate (S3–2, AXTN: acetic acid = 2:1). When 3 g of AXTN and 60 mL of acetic acid were added to the jacketed glass container and stirred with a stirring paddle at a speed of 200 rpm, crystal form IV was created.

In fact, acetonitrile, DMF, and acetic acid solvates have been reported in the literature [30]. More detailed information can be found in Table S2 of the Supplementary Materials. However, the PXRD patterns as well as the stoichiometric ratios of the other solvents, except for the DMF solvate and acetic acid-H_2_O solvate, do not agree with those mentioned in the literature, which are different solvates obtained from the same solvent.

### 3.3. Solid Characterization

#### 3.3.1. Powder X-Ray Diffraction (PXRD)

PXRD patterns were obtained on a Rigaku Mini Flex 600 with Cu Kα radiation (0.15418 nm). During the measurement process, the tube voltage and current are set to 40 kV and 15 mA, respectively. PXRD data were collected on 2θ = 3–50° with a step size of 0.02° and scanning speed of 8° min^−1^.

#### 3.3.2. Differential Scanning Calorimetry

Differential scanning calorimetry (DSC) was performed in the Mettler Toledo DSC 3 STARe system. DSC analysis was calibrated using indium (429.76 ± 0.02 K) and zinc (692.68 ± 0.02 K) [36] (Mettler Toledo, Zurich, Switzerland). The calibration uncertainty is 0.29 K. We weighed 5 mg (with an accuracy of ±0.01 mg) of the sample, placed it in a standard aluminum plate, and then heated it at a scanning rate of 10 K·min^−1^ under a nitrogen flow rate of 50 mL/min.

#### 3.3.3. Thermogravimetric Analysis

Thermogravimetric analysis (TGA) was performed in the Mettler Toledo TGA/DSC3+ STARe system. A total of 5 mg (with an accuracy of ±0.01 mg) of the sample was added to the alumina crucible, and an empty pot was used as a reference. Nitrogen was used as the protective gas. The samples were heated from 303.15 K to 573.15 K with a heating rate of 10 K·min^−1^.

#### 3.3.4. Fourier Transform Infrared (FT-IR) Spectroscopy

FT-IR analysis was carried out using a Vertex 70 FT-IR spectrometer (Bruker, Karlsruhe, Germany). The KBr tableting method was used to prepare the sample. The sample to be tested and KBr were mixed at a mass ratio of 1:50, ground evenly, and pressed into transparent flakes in a tablet press. The spectral resolution was 4 cm^−1^, and the test range was 4000–500 cm^−1^.

#### 3.3.5. Scanning Electron Microscope

A scanning electron microscope (SEM, Hitachi TM4000 II, Tokyo, Japan) was used to observe the difference in surface morphology between solvates and desolvated products. High vacuum was set with an acceleration voltage of 5–15 kV. During the process of sample pretreatment, gold material was used, which sputtered for about 40 s.

### 3.4. Desolvation Experiments

The conversion between solid forms of AXTN (especially solvates) has been studied through solvent removal methods. In this work, methods such as heating, steam-mediated phase transition, microwave treatment, and solvent-mediated phase transition were used to study the desolvation of the AXTN solvates.

#### 3.4.1. Solid-Phase Desolvation Experiments 

Desolvation by heating: First, 0.2 g of the solvate of AXTN was added in a glass dish, which was then placed in a vacuum drying oven at a certain temperature (70 °C or 115 °C). After drying for 2 h, desolvated product was formed. This work also investigated the effect of crystalline seeds on the solid-state phase transition of AXTN solvates. The specific operation is as follows: a certain mass of form A (10% of the mass of the solvate, i.e., 0.02 g) was used as the seeds, which is mixed evenly with the AXTN-acetic acid solvate (S3–2) and heated at 70 °C. Finally, we compared the results obtained with the situation without adding seeds.

Steam-mediated phase transition: The solvate of AXTN was placed in a steam atmosphere of an organic solvent and maintained at 70 °C for 2 h to obtain the desolventized product. The organic solvents (acetonitrile, methanol, or ethanol) were placed in a vacuum drying oven and maintained at 70 °C under vacuum. Under these conditions, the steam atmosphere of the organic solvent was created. The pressure of solvent vapor in the steam was 0.05 MPa.

Desolvation by microwave: The solvate of AXTN was placed in a microwave oven. After microwave treatment for 5 min, the desolvated product of AXTN were formed. The parameters of the microwave oven used in this work were as follows: model, MA-1870M1; output power, 700 W; oscillation frequency, 2450 MHz.

#### 3.4.2. Solvent Mediated Phase Transition (SMPT)

The SMPT experiments were designed to investigate the transformation between the solid-state forms [37,38]. We put an appropriate amount (2 g) of AXTN solvate powder and 15 mL of solvent (acetonitrile, DMF, acetic acid, methanol, ethanol) into a container, and stirred the mixture at 500 rpm to prepare a slurry. The thermostatic water bath was used to maintain the temperature at 40 °C for 6 h, then it was cooled to 20 °C. The solids were collected by filtration and dried at room temperature.

## 4. Conclusions

The crystal form A of AXTN can form solvates in solvents such as acetonitrile, DMF, acetic acid, and methanol. Different ratios of AXTN and acetic acid will form different product (solvate or directly crystallized into another crystal form (form IV)). The obtained solvates were desolvated using methods such as heating, steam-mediated phase transformation, microwave, and solvent-mediated phase transformation. The desolvated samples were characterized by PXRD, TGA, DSC, FT-IR, and SEM. The results indicate that the solvates of AXTN formed a new crystal form (form Z) different from the original crystal form after desolvation. AXTN-acetonitrile solvate tends to transform to form IV after desolvation. After solid-phase desolvation (steam-mediated, heating), form IV is formed in all cases where a phase transition occurs. Compared with other solvates, there are more methods to make AXTN-DMF solvate undergo phase transition, and more types of polymorphs can ultimately be obtained. AXTN-acetic acid solvate can easily produce form VI after desolvation. Methods of solid-phase desolvation other than methanol-steam-mediated phase transformation can enable the transformation of AXTN-acetic acid solvates to form VI. Three different crystal forms of AXTN were obtained under three different solid-phase desolvation methods of AXTN-methanol solvate. Form I was created, which could not be obtained by other solvates in this work. In the process of phase transformation, form VI and form IV are the easily obtained crystal forms. Most solvates will transform into these two crystal forms after desolvation. AXTN-methanol solvate is easily obtained in the SMPT process. Other solvates can be transformed into AXTN-methanol solvate. We also found that if the raw material of AXTN is placed in a mixed solvent with a volume ratio of 1:1, it tends to generate a fixed solvate. Put AXTN into a mixed solvent of methanol/other solvents = 1:1, and AXTN-methanol solvate is formed. This shows that the interaction between methanol molecules and AXTN molecules may be stronger than that of other solvent molecules. This work can provide guidance for polymorph screening, and the new crystal form obtained in this work can provide more choices for AXTN formulations.

## Figures and Tables

**Table 1:** Desolvation methods of AXTN solvates.

Solvate	Method	Abbreviation	Final Form
S1	Methanol steam	E-MeOH	IV
	Ethanol steam	E-EtOH	IV
	Heating at 115 °C	H-115	IV
	Ethanol solvent	S-EtOH	Z
S2	Methanol steam	E-MeOH	IV
	Ethanol steam	E-EtOH	VI
	Heating at 115 °C	H-115	IV
	Microwaving	M	IV
	Ethanol solvent	S-EtOH	A
	Acetic acid solvent	S-HAc	VI
S3–2	Methanol steam	E-MeOH	VI
	Ethanol steam	E-EtOH	IV
	Heating at 70 °C	H-70	VI
	Heating at 70 °C and add seed crystals	H-70seed	VI
	Microwaving	M	VI
S4	Ethanol steam	E-EtOH	VI
	Acetonitrile steam	E-ACN	A
	Heating at 115 °C	H-115	I

**Table 2:** Transformation between solvates of AXTN.

Solvate	Method	Abbreviation	Form
S1	DMF solvent	S-DMF	S2
	Acetic acid solvent	S-HAc	S3–2
	Methanol solvent	S-MeOH	S4
S2	Acetonitrile solvent	S-ACN	S1
	Methanol solvent	S-MeOH	S4
S3–2	DMF solvent	S-DMF	S2
	Methanol solvent	S-MeOH	S4
S4	Acetonitrile solvent	S-ACN	S1
	DMF solvent	S-DMF	S2
	Acetic Acid solvent	S-HAc	S3–2

## Data Availability

The data presented in this study are available in article and Supplementary Materials.

## Supplementary Materials

The following supporting information can be downloaded at: https://www.mdpi.com/article/10.3390/molecules29194696/s1, Table S1: Detailed information of the chemicals used in this work, Table S2: Data have been reported for AXTN solvates, Figure S1: Comparison of the solvates obtained in this work and those that have been reported, Figure S2: Infrared spectra of the raw material and the solvates of AXTN, Figure S3: The SEM images of the raw materials and solvates of AXTN, Figure S4: PXRD patterns of the transformation results between the solvates of AXTN, Figure S5: The conversion relationship between the solvates of AXTN, Figure S6: TGA and DSC curves of form Z.

## References

[1] Duerinck J., Du Four S., Bouttens F., Andre C., Verschaeve V., Van Fraeyenhove F., Chaskis C., D’Haene N., Le Mercier M., Rogiers A. (2018). Randomized phase II trial comparing axitinib with the combination of axitinib and lomustine in patients with recurrent glioblastoma. J. Neuro-Oncol..

[2] Fu Y., Saxu R., Ahmad Ridwan K., Zhao C., Kong X., Rong Y., Zheng W., Yu P., Teng Y. (2022). Selenium substituted axitinib reduces axitinib side effects and maintains its anti-renal tumor activity. RSC Adv..

[3] Kameda T., Takayama T., Sugihara T., Takeshima S., Yamazaki M., Komatsubara M., Kamei J., Fujisaki A., Ando S., Kurokawa S. (2020). The efficacy of axitinib as a first-line treatment for metastatic renal cell carcinoma. Asia Pac. J. Clin. Oncol..

[4] Leslie I., Boos L.A., Larkin J., Pickering L. (2020). Avelumab and axitinib in the treatment of renal cell carcinoma: Safety and efficacy. Expert Rev. Anticancer Ther..

[5] Motzer R.J., Penkov K., Haanen J., Rini B., Albiges L., Campbell M.T., Venugopal B., Kollmannsberger C., Negrier S., Uemura M. (2019). Avelumab plus Axitinib versus Sunitinib for Advanced Renal-Cell Carcinoma. N. Engl. J. Med..

[6] Pemovska T., Johnson E., Kontro M., Repasky G.A., Chen J., Wells P., Cronin C.N., McTigue M., Kallioniemi O., Porkka K. (2015). Axitinib effectively inhibits BCR-ABL1(T315I) with a distinct binding conformation. Nature.

[7] Schmidt D., Rodat T., Heintze L., Weber J., Horbert R., Girreser U., Raeker T., Bußmann L., Kriegs M., Hartke B. (2018). Axitinib: A Photoswitchable Approved Tyrosine Kinase Inhibitor. ChemMedChem.

[8] Spisarová M., Melichar B., Vitásková D., Študentová H. (2021). Pembrolizumab plus axitinib for the treatment of advanced renal cell carcinoma. Expert Rev. Anticancer Ther..

[9] Sonpavde G., Hutson T.E., Rini B.I. (2008). Axitinib for renal cell carcinoma. Expert Opin. Investig. Drugs.

[10] Patson B., Cohen R.B., Olszanski A.J. (2012). Pharmacokinetic evaluation of axitinib. Expert Opin. Drug Metab. Toxicol..

[11] Roberts J.L., Booth L., Poklepovic A., Dent P. (2021). Axitinib and HDAC Inhibitors Interact to Kill Sarcoma Cells. Front. Oncol..

[12] Strosberg J.R., Cives M., Hwang J., Weber T., Nickerson M., Atreya C.E., Venook A., Kelley R.K., Valone T., Morse B. (2016). A phase II study of axitinib in advanced neuroendocrine tumors. Endocr.-Relat. Cancer.

[13] Salama L.B., Duerinck J., Four S.D., Awada G., Fischbuch L., Cremer J.D., Rogiers A., Neyns B. (2018). Safety of axitinib plus avelumab in patients with recurrent glioblastoma. J. Clin. Oncol..

[14] Choueiri T.K., Zakharia Y., Pal S., Kocsis J., Pachynski R., Poprach A., Nixon A.B., Liu Y., Starr M., Lyu J. (2021). Clinical Results and Biomarker Analyses of Axitinib and TRC105 versus Axitinib Alone in Patients with Advanced or Metastatic Renal Cell Carcinoma (TRAXAR). Oncologist.

[15] Pannucci P., March J., Cooper S., Hill S., Woolard J. (2022). Effects of axitinib and lenvatinib on cardiovascular function and haemodynamic. Cardiovasc. Res..

[16] Wei N., Liang J., Peng S., Sun Q., Dai Q., Dong M. (2018). Design, Synthesis, and Biological Evaluation of Axitinib Derivatives. Molecules.

[17] Sedano M.N., Teller J.C., Redondo D.F., Valbuena I.G., Piquero J.F. (2016). CP-136 Effectiveness and safety of axitinib in renal cell carcinoma. Eur. J. Hosp. Pharm..

[18] Heintze L., Schmidt D., Rodat T., Witt L., Ewert J., Kriegs M., Herges R., Peifer C. (2020). Photoswitchable Azo- and Diazocine-Functionalized Derivatives of the VEGFR-2 Inhibitor Axitinib. Int. J. Mol. Sci..

[19] Chen Y., Suzuki A., Tortorici M.A., Garrett M., LaBadie R.R., Umeyama Y., Pithavala Y.K. (2015). Axitinib plasma pharmacokinetics and ethnic differences. Investig. New Drugs.

[20] Chaudhry B., Wu S. (2021). Tolerability of axitinib in advanced renal cell carcinoma: A meta-analysis. J. Clin. Oncol..

[21] Gong J., Zhang D., Ran Y., Zhang K., Du S. (2017). Solvates and polymorphs of clindamycin phosphate: Structural, thermal stability and moisture stability studies. Front. Chem. Sci. Eng..

[22] Gillon A.L., Davey R.J., Storey R., Feeder N., Nichols G., Dent G., Apperley D.C. (2005). Solid State Dehydration Processes:  Mechanism of Water Loss from Crystalline Inosine Dihydrate. J. Phys. Chem. B.

[23] Fujii K., Aoki M., Uekusa H. (2013). Solid-State Hydration/Dehydration of Erythromycin A Investigated by ab Initio Powder X-ray Diffraction Analysis: Stoichiometric and Nonstoichiometric Dehydrated Hydrate. Cryst. Growth Des..

[24] Chakravarty P., Suryanarayanan R. (2010). Characterization and Structure Analysis of Thiamine Hydrochloride Methanol Solvate. Cryst. Growth Des..

[25] Morris K.R., Griesser U.J., Eckhardt C.J., Stowell J.G. (2001). Theoretical approaches to physical transformations of active pharmaceutical ingredients during manufacturing processes. Adv. Drug Delivery Rev..

[26] Singh D., Baruah J.B. (2011). Structural Study on Solvates of Dopamine-Based Cyclic Imide Derivatives. Cryst. Growth Des..

[27] Jørgensen A.C., Strachan C.J., Pöllänen K.H., Koradia V., Tian F., Rantanen J. (2009). An insight into water of crystallization during processing using vibrational spectroscopy. J. Pharm. Sci..

[28] Campeta A.M., Chekal B.P., Abramov Y.A., Meenan P.A., Henson M.J., Shi B., Singer R.A., Horspool K.R. (2010). Development of a Targeted Polymorph Screening Approach for a Complex Polymorphic and Highly Solvating API. J. Pharm. Sci..

[29] Ren B., Dai X., Wang J., Wu C., Lu T., Chen J. (2021). Cocrystallization of axitinib with carboxylic acids: Preparation, crystal structures and dissolution behavior. CrystEngComm.

[30] Qu H., Zhang J., Zhang G., Li Z., Liu Y., Wu S., Gong J. (2022). Structural Insights into the Highly Solvating System of Axitinib via Binary and Ternary Solvates. Cryst. Growth Des..

[31] Huang H., Hu D., Jiang Y., Zhang X. (2014). A New Polymorph of Axitinib.

[32] Burns D.H., Carper W.R. (2004). Anion recognition in a model ion channel: Effects of solvation on chloride binding to a porphyrin–metacyclophane. J. Mol. Struct. THEOCHEM.

[33] Tomasi J., Mennucci B., Cammi R. (2005). Quantum Mechanical Continuum Solvation Models. Chem. Rev..

[34] Mazurek A.H., Szeleszczuk Ł., Pisklak D.M. (2020). Periodic DFT Calculations—Review of Applications in the Pharmaceutical Sciences. Pharmaceutics.

[35] Dubok A.S., Rychkov D.A. (2023). Relative Stability of Pyrazinamide Polymorphs Revisited: A Computational Study of Bending and Brittle Forms Phase Transitions in a Broad Temperature Range. Crystals.

[36] Gatta G.D., Richardson M.J., Sarge S.M., Stølen S. (2006). Standards, calibration, and guidelines in microcalorimetry. Part 2. Calibration standards for differential scanning calorimetry* (IUPAC Technical Report). Pure Appl. Chem..

[37] Shi X., Chen Q., Deng Y., Xing X., Wang C., Ding Z., Su W. (2022). New Case of Pharmaceutical Solid-State Forms: Several Novel Solvates/Polymorphs of Nilotinib and Their Phase Transformation Controls. Cryst. Growth Des..

[38] Hong M., Guo S., Wang K., Ma L., Ren G. (2018). Influence Factor Investigation on the Solution-Mediated Polymorphic Transformation of Baricitinib Phosphate. Z. Anorg. Allg. Chem..

