# Supplementary material for: Solvates and Polymorphs of Axitinib: Characterization and Phase Transformation
Source: Molecules. 2024 Oct 4;29(19):4696. doi: 10.3390/molecules29194696 (PMC11477576; doi:10.3390/molecules29194696)
Supplement: Supplementary file 1 [file molecules-29-04696-s001.zip › molecules-3148155-supplementary.pdf]

Supplementary material

Solvates and Polymorphs of Axitinib: Characterization and Phase transformation

Yinhu Pan[1], Tong Xiao[1], Yan Wang[1], Zhiying Pan[1], Shichao Du[1,*] and Fumin Xue[1,*]

[1] School of Pharmaceutical Sciences (Shandong Analysis and Testing Center), Qilu University of Technology (Shandong Academy of Sciences), Jinan 250014, PR China;

panyinhu98@163.com (Y.P.); 202104350009@stu.qlu.edu.cn (T.X.);

wangyan57@tju.edu.cn (Y.W.); 10431220347@stu.qlu.edu.cn (Z.P.)

** Correspondence: shichao_du@qlu.edu.cn; xuefumin@qlu.edu.cn*

Table S1. Detailed information of the chemicals used in this work.

Chemical name	CAS number	Molar mass ($g.mol^{-1}$)	Source	Mass fraction purity	Analysis method
axitinib	319460-85-0	386.47	Shandong New Era Pharmaceutical Co., Ltd.	≥ 0.99	HPLC[a]
ethanol	64-17-5	46.07	Sinopharm Chemical Reagent Co., Ltd.	≥ 0.997	GC[b]
methanol	67-56-1	32.042	Sinopharm Chemical Reagent Co., Ltd.	≥ 0.99	GC[b]
N,N-dimethyl-formamide (DMF)	68-12-2	73.095	Sinopharm Chemical Reagent Co., Ltd.	≥ 0.995	GC[b]
acetonitrile	75-05-8	41.052	Sinopharm Chemical Reagent Co., Ltd.	≥ 0.99	GC[b]
acetic acid	64-19-7	60.052	Sinopharm Chemical Reagent Co., Ltd.	≥ 0.99	GC[b]

[a]High performance liquid chromatography. [b]Gas chromatography. Both the analytical methods were provided by the suppliers.

Table S2. Data have been reported for AXTN solvates

CSD code	solvent	ratio (AXTN: solvent)
QARTUC	acetonitrile	1:1
QARVAK	DMF	1:1
QARVEO	acetic acid	1:3
QARVIS	acetic acid + H_2O	2:3:1.5

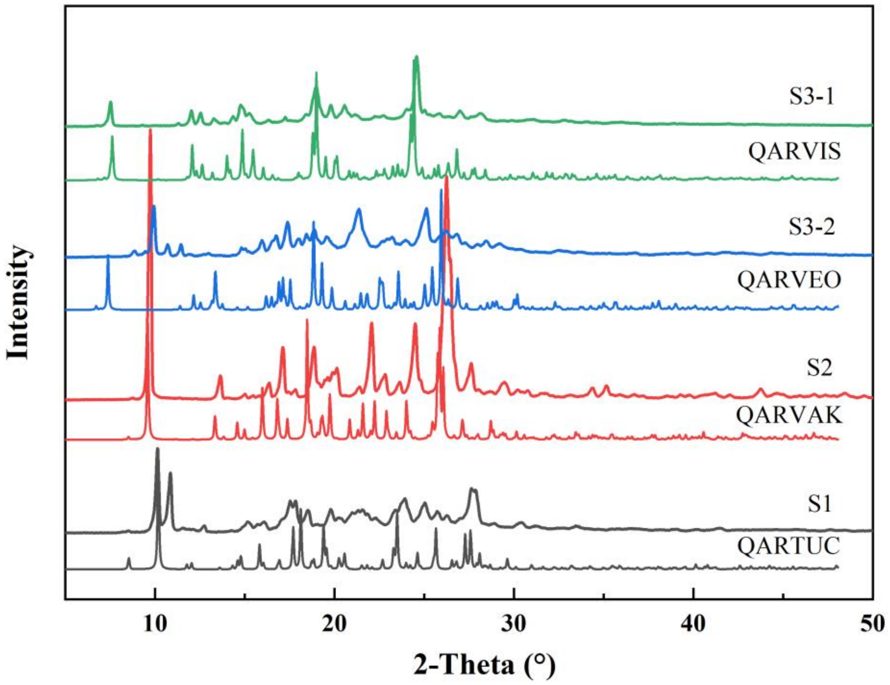

Figure S1. Comparison of the solvates obtained in this work and those that have been reported

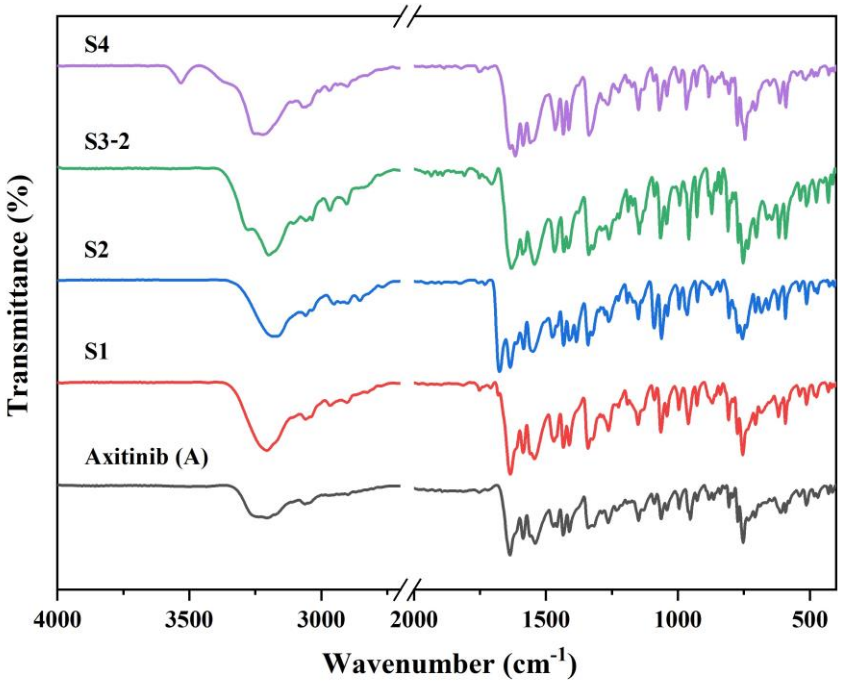

Figure S2. The infrared spectra of the raw material and the solvates of AXTN

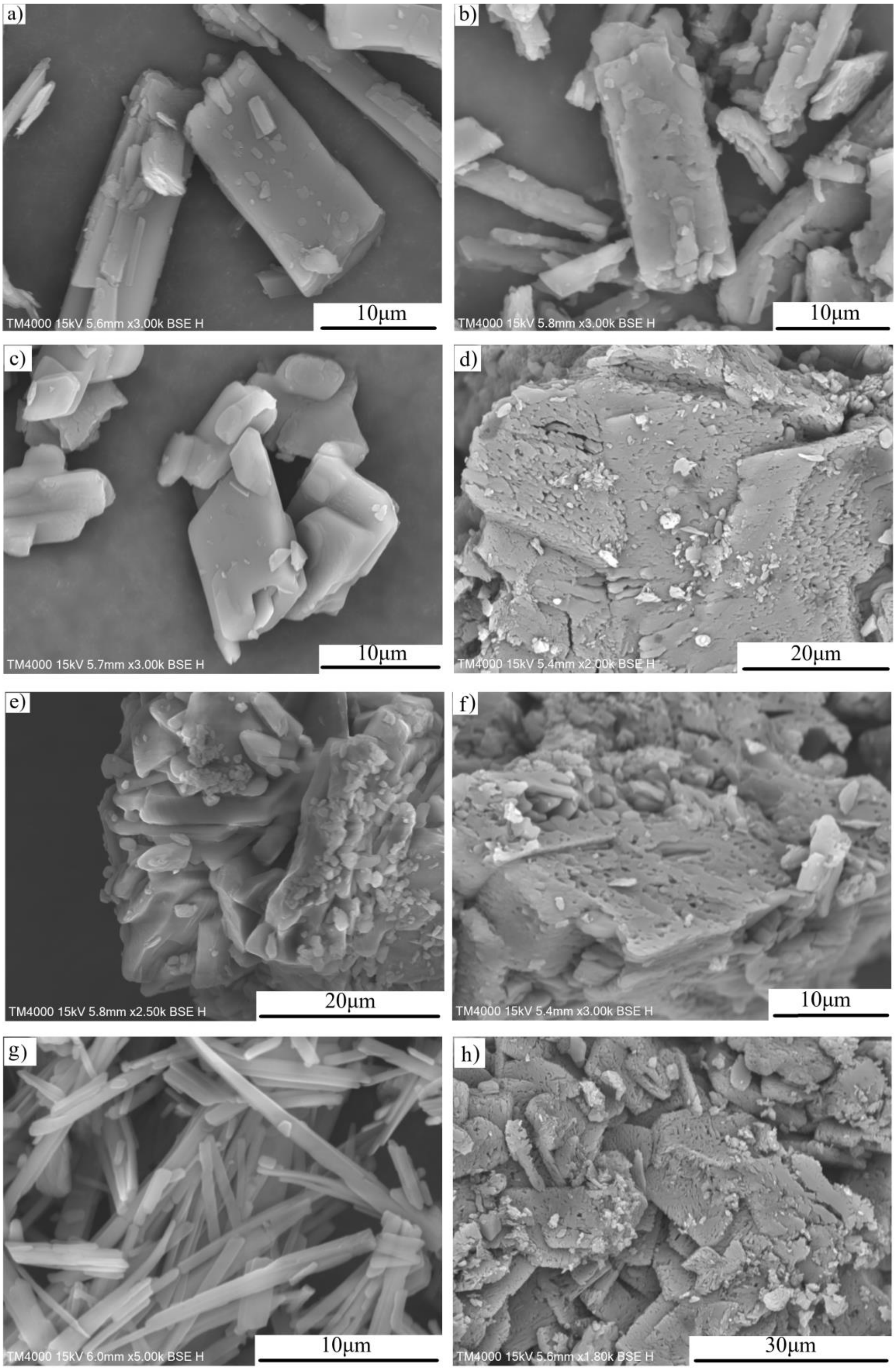

Figure S3. The SEM images of the raw materials and solvates of AXTN

a) The SEM image of the solvate of AXTN-acetonitrile (S1, 3000 x)

b) The product of S1 after methanol steam-mediation (3000 x)

c) The SEM image of the solvate of AXTN-DMF (S2, 3000 x)

d) The product of S2 after methanol steam-mediation (2000 x)

e) The SEM image of the solvate of AXTN-acetic acid (S3-2, 3000 x)

f) The product of S3-2 after methanol steam-mediation (3000 x)

g) The SEM image of the solvate of AXTN-methanol (S4, 3000 x)

h) The product of S4 after acetonitrile steam-mediation (1800 x)

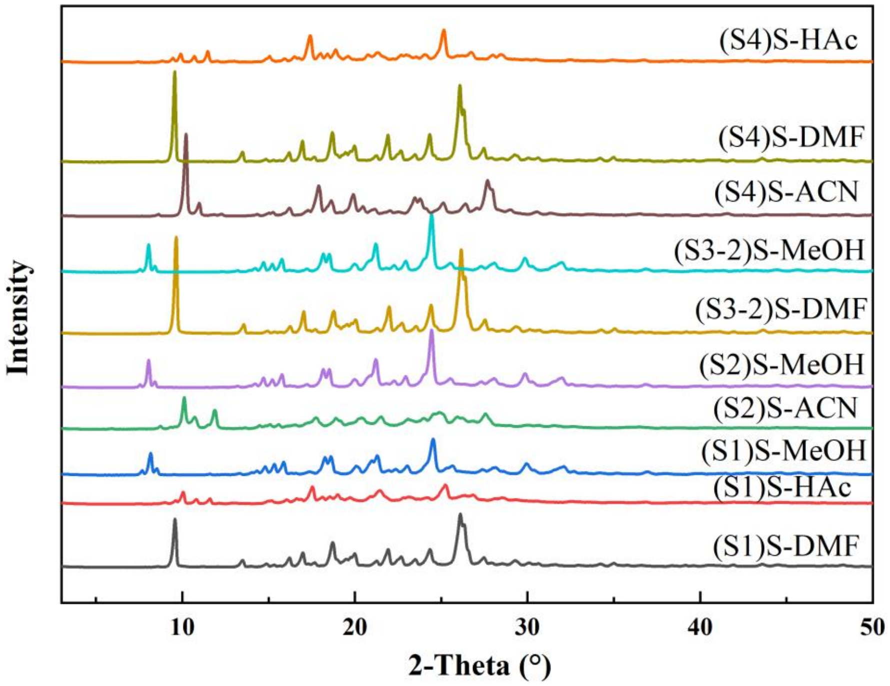

Figure S4. The PXRD patterns of the transformation results between the solvates of AXTN

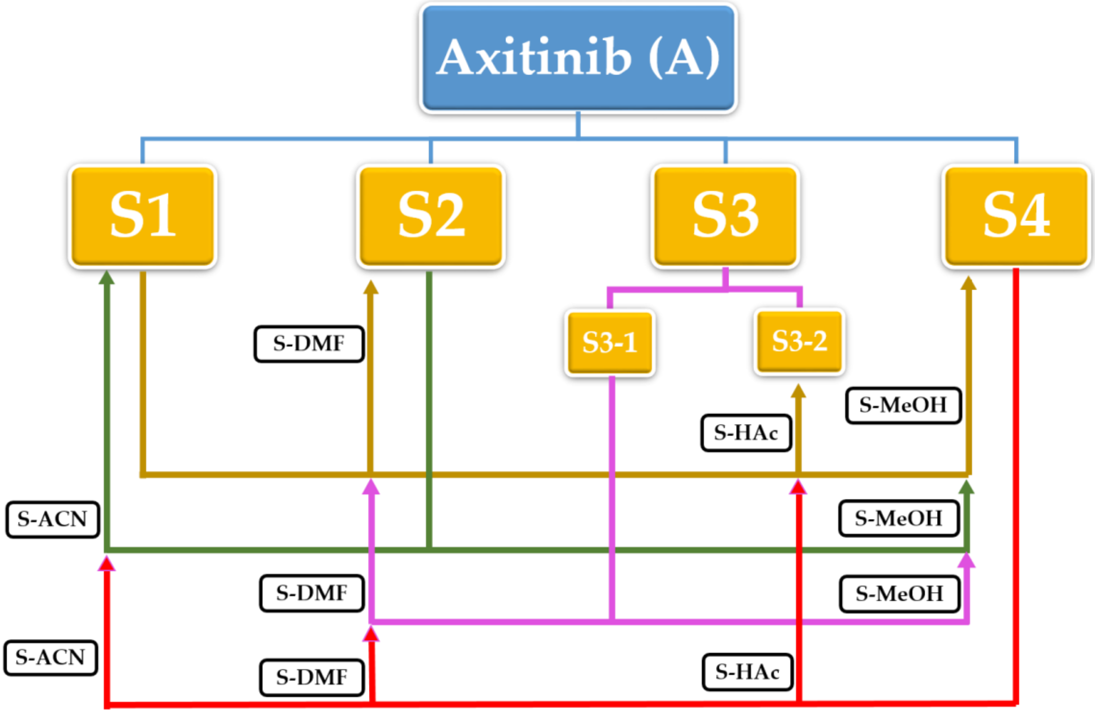

Figure S5. The conversion relationship between the solvates of AXTN.

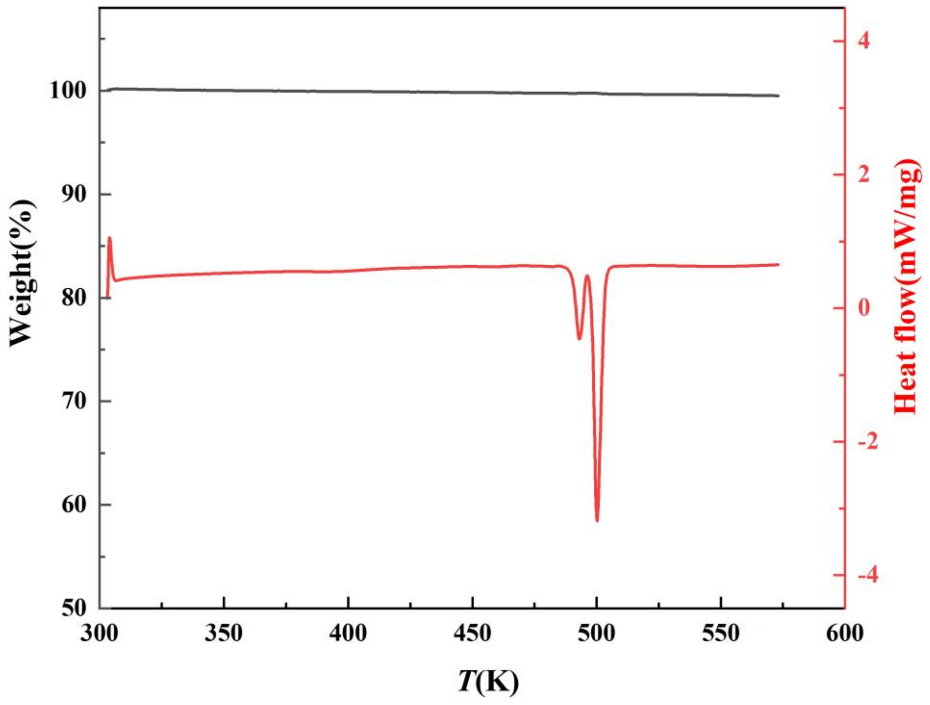

Figure S6. TGA and DSC curves of form Z
